# The impact of supplementation with pomegranate fruit (*Punica granatum* L.) juice on selected antioxidant parameters and markers of iron metabolism in rowers

**DOI:** 10.1186/s12970-018-0241-z

**Published:** 2018-07-24

**Authors:** A. Urbaniak, P. Basta, K. Ast, A. Wołoszyn, J. Kuriańska – Wołoszyn, Ewa Latour, A. Skarpańska – Stejnborn

**Affiliations:** 1 Department of Morphological and Health Sciences, Faculty of Physical Culture in Gorzów Wlkp. Poland, 13 Estkowskiego Str., 66 – 400 Gorzów Wlkp, Poland; 2 Department of Water Sports, Faculty of Physical Culture in Gorzów Wlkp. Poland, 13 Estkowskiego Str., 66 – 400 Gorzów Wlkp, Poland; 3 Jacob of Paradies University in Gorzów Wielkopolski, Teatralna Str. 25, Gorzów Wielkopolski, 66-400 Poland; 4 Faculty of Physical Culture in Gorzów Wlkp, 13 Estkowskiego Str., 66 – 400 Gorzów Wlkp, Poland

**Keywords:** Pomegranate, Strenuous exercise, Training, Inflammation

## Abstract

**Background:**

The aim of this study was to analyse the effect of pomegranate juice (POM) supplementation on the levels of selected pro-inflammatory cytokines, hepcidin and markers of iron metabolism in well-trained rowers.

**Method:**

The double-blind placebo-controlled study included 19 members of the Polish Rowing Team. The athletes were randomised into the supplemented group (*n* = 10), receiving 50 ml of standardised POM daily for two months, or the placebo group (*n* = 9). The subjects performed a 2000 m test on the rowing ergometer at the start of the project (baseline) and end of follow-up period. Blood samples from the antecubital vein were obtained three times during each trial: prior to the exercise, one minute after the test, and following a 24 h recovery.

**Results:**

The study documented the beneficial effect of supplementation with pomegranate fruit juice on TAC *(P < 0.002)*. During the resting period, TAC level in the supplemented group was significantly higher than in the placebo group (x ± SD, 2.49 ± 0.39 vs. 1.88 ± 0.45, *P < 0.05*). The ergometric test conducted at baseline demonstrated a significant post-exercise increase in the concentrations of soluble transferrin receptors *(P < 0.04)*, iron *(P < 0.002)* and IL-6 *(P < 0.02)*, and to a significant post-exercise decrease in TAC. A significant increase in IL-6 concentration was also observed 24 h post-exercise. The exercise test conducted at the end of the follow-up period resulted in a significant decrease in TBIC and a significant increase in UIBC *(P < 0.001)*, observed in both groups, both immediately post-exercise and after the resting period.

**Conclusion:**

Supplementation with POM contributed to a significant strengthening of plasma antioxidant potential in the group of well-trained rowers, but had no effect on iron metabolism markers.

## Background

Physical training, in particular strenuous exercises, places a considerable burden on an athlete’s body. To achieve outstanding results in competitive sports, athletes need to bear large, and not infrequently extreme, training loads. This can result in a disruption to their intrinsic homeostasis, and thus have an unfavourable effect on their performance. Published evidence suggests that a key determinant of athletes’ performance is iron metabolism [1, 2]. Normal levels of iron are a prerequisite for many physiological processes, such as oxygen transport and energy synthesis [3].

The level of iron in the human body is affected not only by an adequate dietary intake of this element, but also by exercise-induced inflammation [4]. The discovery of hepcidin, a hormone, provided the link between iron deficiency and concomitant inflammation [5]. Studies have demonstrated that moderate-intensity training has a beneficial effect on iron metabolism, whereas strenuous exercise may induce systemic inflammation [6]. The inflammatory response is associated with enhanced synthesis of hepcidin; this hormone is involved in the degradation of ferroportin, and thus prevents the mobilisation of iron from its cellular deposits (e.g. in the liver and spleen) and interferes with its gastrointestinal absorption [7]. If this state persists, it may negatively affect erythrocyte parameters, and eventually lead to anaemia [8]. Reinke et al. [9] showed that at the end of competitive season, 70% of professional athletes (rowers and football players) presented with functional iron deficiency, and 27% had an absolute deficiency of this element. Unfavourable changes in iron parameters after strenuous exercise have also been reported by other authors [10].

Additional supplementation with iron-rich preparations does not necessarily bring expected results, and may be even harmful to an athlete’s health [11, 12]. In an animal study conducted by Reardon et al. [13], injection of iron contributed to an increase in muscle and plasma concentrations of this element and to exacerbation of oxidative stress; as a result, animals from iron-supplemented group presented with lower muscle power than the controls and performed less work during the treadmill test.

The post-exercise increase in hepcidin level has been also postulated to result from an increase in the so-called labile iron pool (LIP) [1], caused by enhanced haemolysis [14]. Products of heme degradation may stimulate the generation of reactive oxygen species, leading to further damage of erythrocyte membranes. The latter are abundant in polyunsaturated fatty acids, which makes them particularly prone to oxidative injury. This self-perpetuating process may last as long as its natural “attenuation” occurs.

Pomegranate juice (POM) from the fruits of *Punnica Granatum* L. is a rich source of polyphenols, such as anthocyanins, flavanols and some ellagitannins, especially punicalagin [15]. Many studies have documented the beneficial effects of POM consumption in the treatment of various disorders [16, 17]. Researchers have recently become increasingly interested in the dietary supplementation of athletes with POM. Fuster-Mũnoz et al. [18] demonstrated that POM exerted a positive effect on the modulation of fat and protein damage in well-trained endurance-based athletes. Ammar et al. [19] showed that the consumption of POM 48 h prior to, and during training sessions contributed to the alleviation of pain, delayed damage, inflammation, and soreness of the knee flexor, accelerated the recovery kinetics of biological parameters and improved performance in nine elite weightlifters.

One group of polyphenols in pomegranate juice are anthocyanins. These compounds have an array of biological activities, showing antioxidant properties [20], acting as immunostimulants, modulating inflammatory response [21] and chelating iron ions, which may contribute to the reduction of LIP [22].

We hypothesised that supplementation with pomegranate fruit juice may boost the antioxidant potential of the athletes, contributing to an increase in TAC, and may thus attenuate the inflammatory response triggered by intense physical exercise. We also examined whether, and to what extent, these changes affected iron metabolism parameters in the study subjects.

## Methods

The protocol of the study was approved by the local bioethics committee at the University of Medical Sciences in Poznan (Decision no. 357/15). All athletes were adequately informed about the nature of the study and provided their written consent to participate in the project.

### Participants

The study included a group of 19 male rowers, members of the Polish National Team, who participated in an eight-week training camp between the preparatory and competitive periods. The basic characteristics of the study athletes are shown in Table 1. The study subjects were randomised to one of two groups, receiving standardised POM (supplemented group, *n* = 10) or a placebo (control group, *n* = 9).

**Table 1:** Basic characteristics of the study groups (mean ± standard deviation)

Parameter	Supplemented group (*n* = 10)	Control group (*n* = 9)
Age (years)	20.8 ± 0.86	20.9 ± 0.95
Body weight (kg)	89.4 ± 8.97	83.85 ± 12.04
Body height (cm)	192.1 ± 6.64	189.6 ± 5.79
Duration of training (years)	8.2 ± 0.78	7.14 ± 0.69

### Food intake

Rowers from the supplemented group received 50 ml of a standardised, commercially available POM (Oleofarm®) daily, for two months. The product was 100% pure natural juice squeezed from fresh fruit, with a total polyphenol content equal to 220 mg/100 g. Athletes from the non-supplemented group received the same dose of a placebo composed of water, sugar and grenadine, with a colour and taste resembling that of the pomegranate fruit juice. Both pomegranate fruit juice and placebo were provided by the same manufacturer (Oleofarm®), packed in identical dark bottles labelled with encoded information about the type of preparation and its recommended dosage. The labels were decoded at the end of the study.

The athletes completed food intake questionnaires on each day of the study period, which were later used to calculate the energy equivalents for their diet and the dietary intake of antioxidants and vitamins. All study subjects agreed that they refrained from drugs, medications and dietary supplements for at least two weeks preceding the study and throughout the whole study period.

### Experimental procedure

The characteristics of training profiles, such as intensity, volume (in min) and type (specific, i.e. rowing: endurance, technical, speed, etc., and non-specific: jogging, strength) were recorded on a daily basis. Training intensity was classified in relation to the lactic acid (LA) threshold (4 mmol/L), as an extensive (below the LA threshold) or intensive (above the LA threshold) workload (Table 2). On the first day (prior to supplementation, at the baseline) and at the end of the training camp (after supplementation, at the end of the follow-up period), the athletes performed a controlled 2000-m rowing exercise test (Concept II, Model D, USA). All study subjects were asked to perform both tests at their maximal pace. Each test was preceded by a five minute warm-up session.

**Table 2:** Training schedules during the weeks preceding blood samples before (Trial I) and after (Trial II) the supplementation

	Days before blood sample collection						
	1	2	3	4	5	6	7
Total training time [min/day]	120	100	200	190	210	150	120
Time rowed [min/day]	110	100	100	100	70	90	100
Distance rowed [km/day]	22	20	20	20	16	18	20
Training for force development [min/day]	–	–	90	–	70	–	–
Extensive endurance rowing training time [min/day]	70	100	100	40	40	90	100
High-intensity endurance rowing training time [min/day]	40	–	–	60	30	–	–
Unspecific training (running etc.) [min/day]	10	–	10	90	70	60	20
Total training time [min/day]	180	100	190	160	200	90	130
Time rowed [min/day]	160	100	130	140	120	90	125
Distance rowed [km/day]	32	18	26	28	20	16	20
Training for force development [min/day]	–	–	60	–	60	–	–
Extensive endurance rowing training time [min/day]	160	100	130	90	94	90	125
High-intensity endurance rowing training time [min/day]	–	–	–	30	26	–	5
Unspecific training (running etc.) [min/day]	20	–	25	20	20	–	–

Blood samples were collected for the analysis (0.9 ml) prior to each 2000 m test (in the morning, after an overnight fasting), one minute after the test and following a 24 h recovery period. Immediately after collection, the samples were centrifuged to separate erythrocytes from serum. The serum was immediately frozen and stored at − 80 °C until the analysis. Capillary blood samples were obtained via an ear lobe prick before and after each exercise test, to assess LA levels.

### Measurements

Total antioxidant capacity (TAC), was measured as an indicator of plasma antioxidant capacity with a commercially available ELISA kit (Cayman, cat no. Antioxidant Assay 709,001–96, USA); the results were expressed in mmol/L. Uric acid (UA) level was determined with a commercially available kit (Alpha Diagnostics, Cat No. K6681–100); the results were expressed in mg/dL. Serum interleukin 6 (IL-6) was quantified with a commercially available enzyme-linked immunosorbent assay (ELISA; Quantikine HS, R&D Systems, Minneapolis, USA); the results were expressed in pg/ml. Serum hepcidin was measured using a commercially available ELISA kit (Wuhan EIAab Science Co., China); the results were expressed in ng/mL. Iron concentration and TIBC were determined using colorimetric method with chromogens (cat. no. 1–418–01-50; 1–421-0060 BioMaxima, Poland); the results were expressed in μg/dL. Unsaturated iron-binding capacity (UIBC) was calculated from the formula: UIBC = TIBC – Fe. Myoglobin concentration was determined immunochemically, using Myoglobin ELISA kit (Biocom, cat. no. 11170); the results were expressed in ng/mL. Serum ferritin was quantified immunochemically, with a commercially available diagnostic kit (Demeditec, Germany); the results were expressed in ng/ml. Concentration of soluble transferrin receptor (sTfR) was determined immunochemically, with a commercially available diagnostic kit (Biocom, cat. no. RD194011100); the results were expressed in μg/mL. Creatine kinase (CK) activity in blood plasma was determined with a commercially available kit (Dr Lange, Germany, cat. no. LCN 282); the results were expressed in U/L. The coefficients of variation for all assays were < 12%.

The concentration of LA in capillary blood was measured immediately after sampling, with a commercially available kit (cat. no. LKM 140, HACH LANGE, Düsseldorf, Germany); the results were presented in mmol/L.

### Statistical analysis

Statistical analysis of the results was carried out with the STATISTICA v. 10.0 software package (StatSoft, Cracow, Poland). The significance of intergroup and intragroup differences was verified using 2 (supplemented and placebo group) × 3 (timing of measurement) repeated measures analysis of variance (ANOVA). Normal distribution of the study variables was verified with a Shapiro-Wilk test. When statistically significant differences were documented on ANOVA, Fisher’s *post-hoc* tests were conducted to identify the source of variance. The anthropometric characteristics of the study groups were compared with unpaired Student *t*-tests. Except for the rowing time, the results of the 2000 m tests performed prior to and after the supplementation period were subjected to intragroup and intergroup comparisons with paired and unpaired Student *t*-tests, respectively. The results of the 2000 m simulated rowing test were subjected to one-way ANOVA. The statistical characteristics of the study variables are presented as arithmetic means ± standard deviations (SD). The threshold of statistical significance for all tests was set at *p* < 0.05.

## Results

**Fig. 1:**
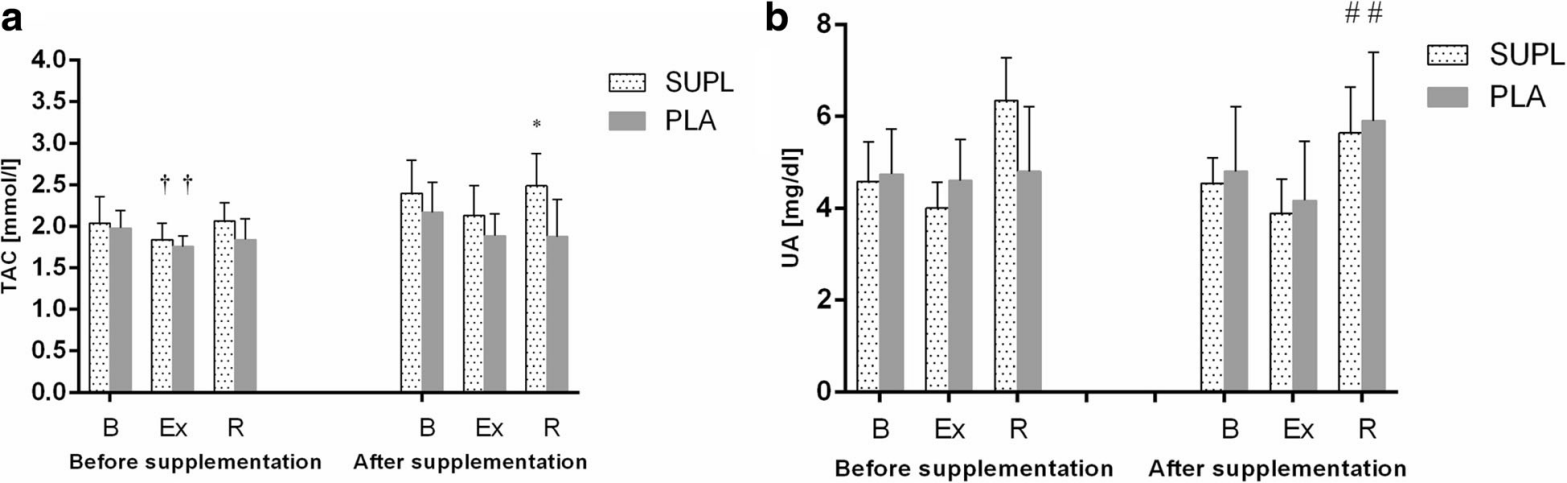
Data represents the mean (SD) values for total antioxidant capacity (TAC) (**a**) and uric acid (UA) (**b**) levels during exercise tests performed before and after supplementation (mean ± SD). Note: TAC = total antioxidant capacity; UA = uric acid; - SUPL = supplemented group; - PLA = placebo group; B = baseline; Ex = immediately after the exercise; R = after a 1-day recovery; † − significantly different compared to baseline level; * - significantly different compared to PLA; # - significantly different compared to post-exercise level

**Fig. 2:**
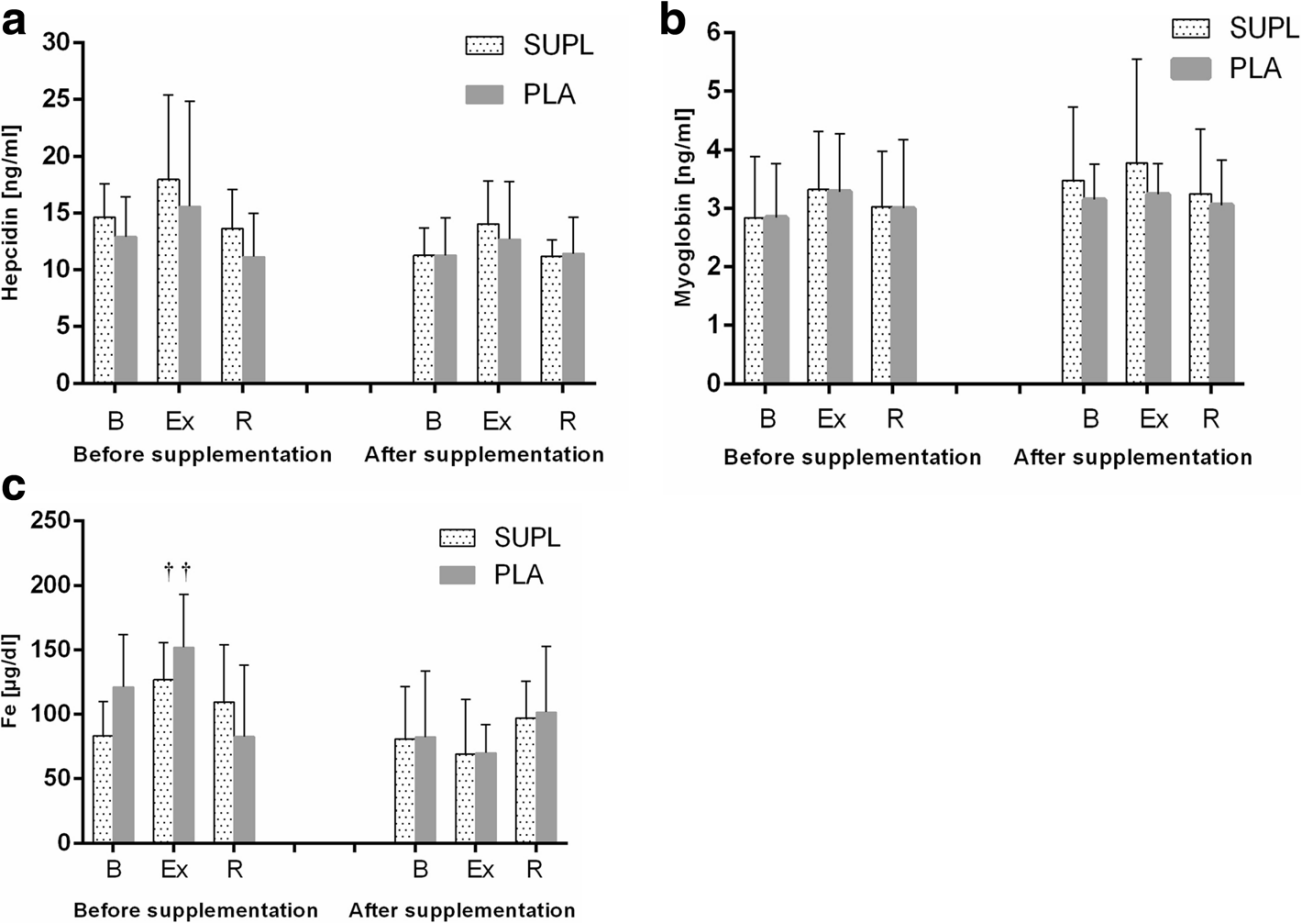
Data represents the mean (SD) values for hepcidin (**a**), myoglobin (**b**) and iron (Fe) (**c**) levels during exercise tests performed before and after the supplementation (mean ± SD). Note: Fe = iron; - SUPL = supplemented group; - PLA = placebo group; B = baseline; Ex = immediately after the exercise; R = after a 1-day recovery; † − significantly different compared to baseline level

**Fig. 3:**
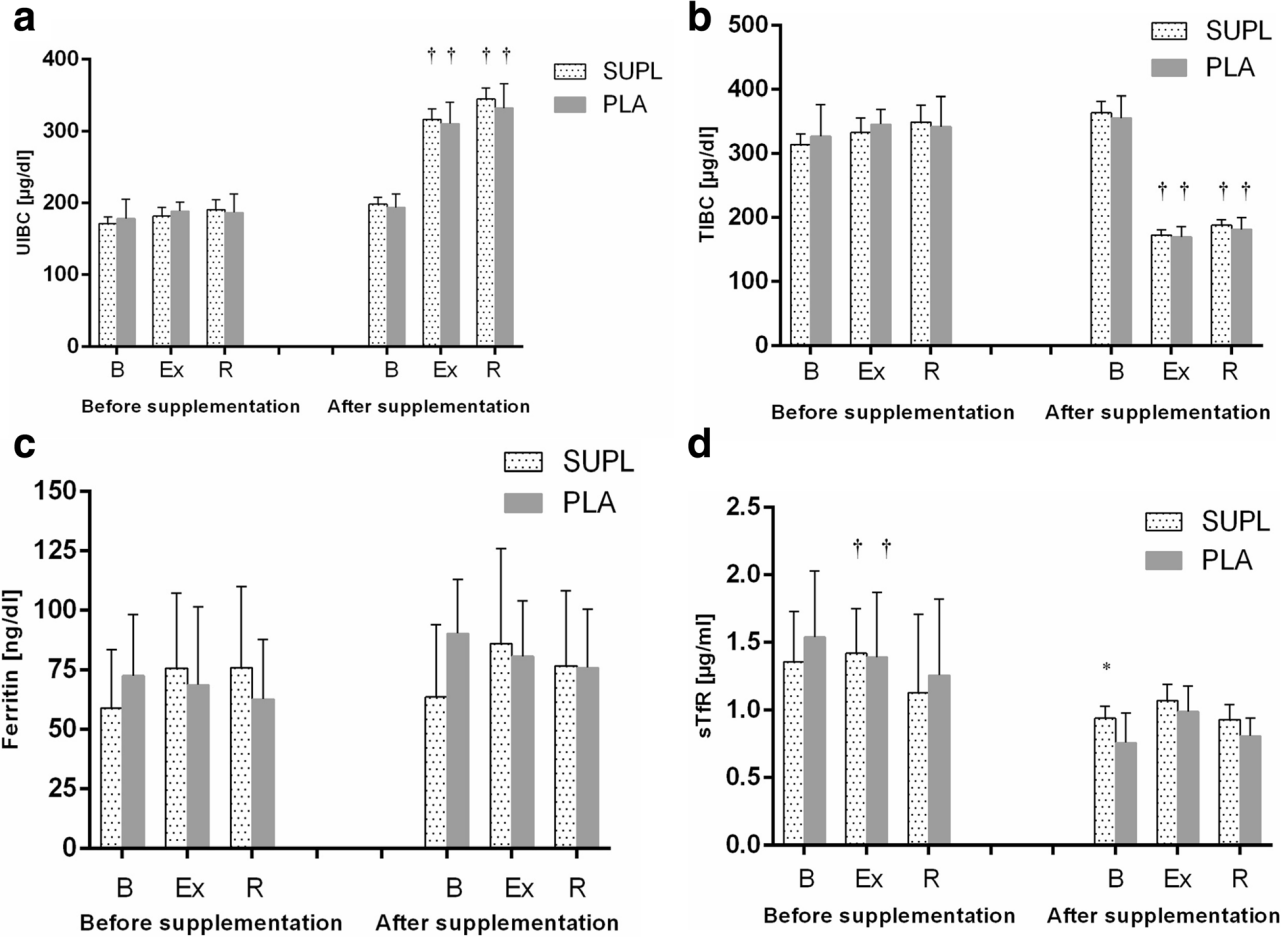
Data represents the mean (SD) values for iron binding capacity (UIBC) (**a**), total iron binding capacity (TIBC) (**b**), ferritin (**c**) and soluble transferrin receptors (sTfR) (**d**) levels during exercise tests performed before and after the supplementation (mean ± SD). Note: UIBC = unsaturated iron binding capacity; TIBC = total iron binding capacity; sTfR = soluble transferrin receptors;- SUPL = supplemented group; - PLA = placebo group; B = baseline; Ex = immediately after the exercise; R = after a 1-day recovery; † − significantly different compared to baseline level; * - significantly different compared to PLA

**Fig. 4:**
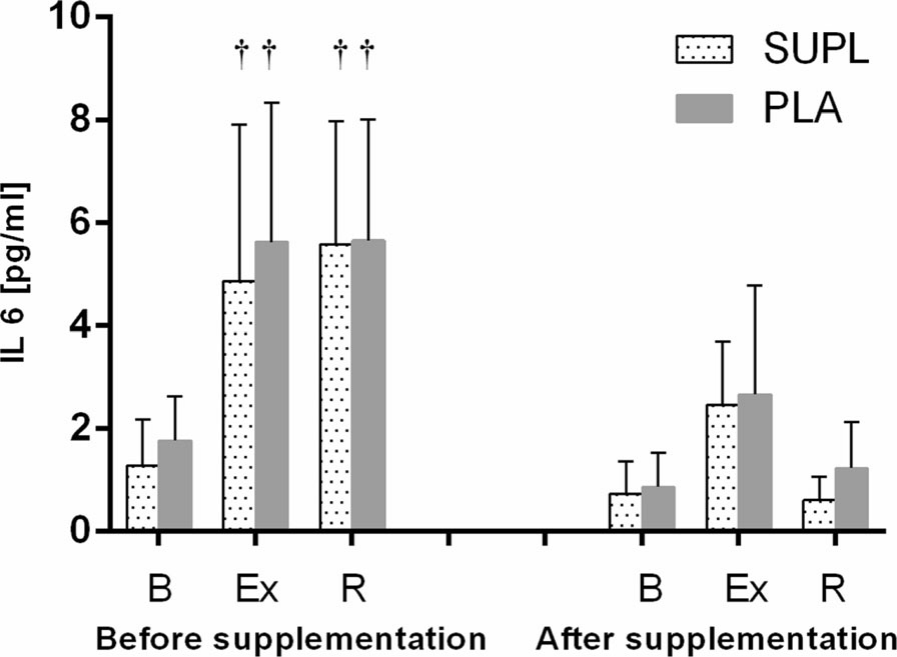
Data represents the mean (SD) values for interleukin 6 (IL-6) levels during exercise tests performed before and after the supplementation (mean ± SD). Note: IL 6 = interleukin 6; - SUPL = supplemented group; - PLA = placebo group; B = baseline; Ex = immediately after the exercise; R = after a 1-day recovery; † − significantly different compared to baseline level

Athletes from the supplemented group did not differ significantly from the controls in terms of their anthropometric parameters, age and training experience (Table 1). No statistically significant intergroup differences were found in power output, total row time over a 2000 m distance, or pre- and post-test LA levels (Table 3). The TAC values were determined prior to and after the supplementation period, and are presented in Fig. 1a. TAC turned out to be modulated by both physical exercise (*p* < 0.029) and POM supplementation (*p* < 0.002). In the baseline measurements, athletes from both study groups showed a post-exercise decrease in TAC levels. At the end of the follow-up, post-recovery TAC level in athletes from the supplemented group was significantly higher than in the controls. Regardless of the study group, post-recovery UA concentrations at baseline were significantly higher (*p* < 0.0004) than those determined immediately after the exercise test (Fig. 1b). Supplementation with POM had no significant effects on serum hepcidin, myoglobin or creatine kinase levels (Fig. 2a, b, d). Athletes from both groups showed a significant post-exercise increase in iron level (*p* < 0.002, Fig. 2c) at baseline measurements. Statistically significant changes in UIBC and TIBC were observed in both study groups, but were statistically significant post-intervention (Fig. 3a and b respectively). UIBC increased immediately after the exercise test (*p* < 0.001) and remained elevated until the end of the recovery period. The exercise test contributed to a significant decrease in TIBC level (p < 0.001), which persisted at the end of the recovery period. ANOVA did not demonstrate the significant effect of POM supplementation on any of these parameters. None of the study groups showed significant changes in ferritin levels at any point of the study (Fig. 3c). The results for sTfR are presented in Fig. 3d. A significant post-exercise increase in sTfR was observed at baseline in the supplemented group (*p* < 0.04), along with a significant decrease in this parameter in the controls. Post-intervention, athletes from the supplemented group presented with significantly higher pre-exercise levels of sTfR than the controls (*p* < 0.012). Pre- and post-supplementation changes in IL-6 levels are shown in Fig. 4. As demonstrated in the ANOVA, physical exercise had a significant effect on the concentration of this cytokine (*p* < 0.02). Prior to the supplementation period, athletes from both groups showed a significant post-exercise increase in IL-6 levels, which persisted post-recovery. This effect was no longer observed at the end of the follow-up period.

**Table 3:** Changes in a 2000 m rowing ergometer performance before and after the supplementation

Parameters	Supplemented group (*n* = 10)		Control group (*n* = 9)	
	Before	After	Before	After
Power (watt)	432 ± 38.4	439 ± 36.4	424 ± 41.3	430 ± 45.6
(W/kg)	4.92 ± 0.29	4.93 ± 0.33	4.96 ± 0.24	5.03 ± 0.20
LA_min_ (mmol/L)^a^	1.8 ± 0.48	1.5 ± 0.26	1.8 ± 0.25	1.6 ± 0.23
LA_max_ (mmol/L)^a^	15.2 ± 2.72	16.33 ± 4.11	13.0 ± 1.84	14.44 ± 2.97
Time (s)	373 ± 11.5	371 ± 10.3	375 ± 12.02	378 ± 15.56

Values represent mean ± standard deviation. No significant differences were found between pre- and post-supplementation parameters (*p* < 0.05)

^a^LA lactic acid

## Discussion

In this study, supplementation with pomegranate fruit juice boosted the antioxidant potential of rowers, as expressed by TAC. The level of this parameter in the supplemented group was significantly higher during the restitution period than in the placebo group (Fig. 1a). The increase in antioxidant potential did not exert a significant effect on other study parameters, however. Previous studies [23] have demonstrated that pomegranate fruit juice has three-fold greater antioxidant activity than other food products widely recognised for their antioxidant properties, such as red wine and green tea. The antioxidant potential of pomegranate fruit juice results from its high content of polyphenols, especially proanthocyanidins [24]. An increase in TAC after a two week supplementation with pomegranate fruit juice has also been reported by other authors [25].

Prior to the supplementation (at baseline), intense physical exercise resulted in a significant decrease in TAC in the study athletes (Fig. 1). Free radicals that are accumulated in excess and inadequately inactivated may, inter alia, initiate the peroxidation of polyunsaturated fatty acids of erythrocyte membranes, and thus enhance post-exercise haemolysis [26, 27]. This hypothesis might also be supported by the observation that prior to supplementation, our rowers showed greater post-exercise increases in iron concentration (Fig. 2c). Manthou et al. [28] demonstrated that healthy subjects supplemented for 14 days with pomegranate fruit juice had increased RBC count, haemoglobin concentration and haematocrit levels. According to those authors, these favourable changes might result from more effective prevention of RBC degradation among other thinks. Fiorani et al. [29] demonstrated that human erythrocytes can absorb extracellular flavonoids via passive diffusion, and constitute a reservoir of these compounds. While most flavonoids (according to the authors, up to 85%) reach the cytosol, some are incorporated into cell membrane. Studies [30, 31] have shown that, similar to cholesterol and alpha-tocopherol, intracellular flavonoids are localised in close proximity to the cell membrane, between the lipid bilayer and aqueous phase. As a result of this location, flavonoids play a vital role in the cell, stabilising plasma membranes that become less fluid, and thus, more resistant to oxidation [32]. Another key issue is cooperation between flavonoids, alpha-tocopherol and ascorbic acid. Flavonoids were shown to inhibit the oxidation of intracellular alpha-tocopherol and to regenerate (as does vitamin C) oxidised alpha-tocopherol to its radical. Ascorbic acid, also protected by flavonoids against oxidation, can in turn inhibit oxidative changes in flavonoids, prolonging their protective effect [33, 34]. Flavonoids therefore maintain a relative balance between oxidised and reduced forms of antioxidants and their radicals, and therefore provide another protective mechanism against elevated concentrations of reactive oxygen species.

Although only athletes from the supplemented group presented with enhanced antioxidant potential during the ergometric test conducted at the end of the follow-up period, physical exercise did not induce significant changes in TAC in either study group (Fig. 1a). Uric acid, the final product of purine metabolism, which proved to be an important antioxidant of blood plasma during in vivo studies [35], did not contribute to changes in TAC levels, although our rowers presented with elevated concentrations during the restitution period (Fig. 1b). Braakhuis et al. [36] demonstrated that the result of a 30 min rowing-ergometer test correlated positively with years and hours of training and the antioxidant status of the blood in elite rowers. According to those authors, these factors had a greater impact on TAC than the dietary intake of antioxidants. The results of our present study suggest that another modulator of TAC may be the phase of the training cycle. The second ergometric test took place during the competitive period when the organism of a well-trained athlete should be characterised by so-called “readiness for competition”, that is be fully adapted to an exercise load specific for a given discipline. It should be stressed that during rowing competitions, athletes participate in qualification and final races, and sometimes need to cover a 2000 m distance twice in a single day. The adaptation of our rowers to this type of exercise load was confirmed by other parameters analysed: a lack of statistically significant changes in IL-6 concentration (Fig. 4) and post-exercise increases in iron levels (Fig. 2c). A study of elite male rowers conducted prior to the Rowing World Championships showed a significant association between the level of proinflammatory cytokines, such as IL-1β, TNF-α and IL-6, and measures of depressed mood, sleep disturbances and fatigue [37]. The lack of statistically significant post-exercise changes in concentrations of proinflammatory cytokines may thus provide important information about the readiness of athletes for competition.

**Fig. 5:**
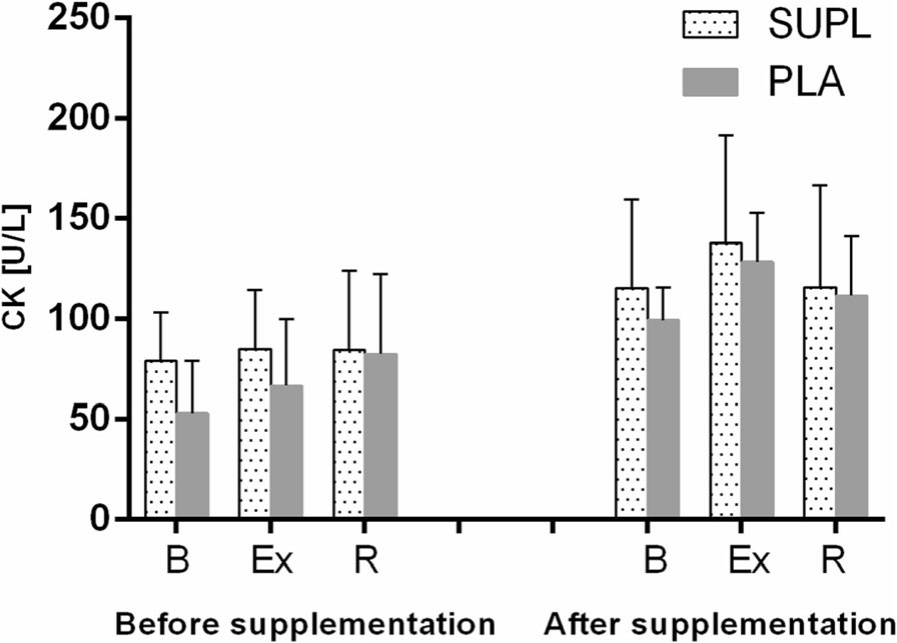
Data represents the mean (SD) values for creatine kinase (CK) levels during exercise tests performed before and after the supplementation (mean ± SD). Note: CK = creatine kinase;- SUPL = supplemented group; - PLA = placebo group; B = baseline; Ex = immediately after the exercise; R = after a 1 – day recovery

Irrespective of the testing period, our athletes did not show statistically significant changes in hepcidin, myoglobin or CK levels (Fig. 2a, b, Fig. 5). To the best of our knowledge, CK activity has rarely been studied in POM-supplemented subjects. We found only one report documenting a significant increase in CK activity in a group of recreationally active males receiving either POM or a placebo for a period of nine days; this effect was probably a consequence of myocyte damage in both study groups [38]. The lack of significant changes in hepcidin, myoglobin and CK levels in our study subjects could perhaps be explained by their good adaptation to large training loads; this issue seems to be an interesting topic for future research. Nevertheless, athletes from both groups showed a significant post-exercise increase in serum concentration iron at baseline measurements (Fig. 2c).

Both supplemented athletes and controls showed a significant post-exercise increase in UIBC during the follow-up test, which persisted after a 24 h recovery (Fig. 3a). Similarly, the post-exercise changes in TIBC seemed to be supplementation-independent, since a significant post-exercise decrease in this parameter was observed post-intervention regardless of the study group, both immediately after the ergometric test and following a 24 h recovery (Fig. 3b). Monitoring of sTfR and body iron has previously shown to be a reliable tool for the determination of Fe metabolism and successful prevention of its deficiency [39]. In our present study, ergometric tests conducted at the baseline contributed to a significant increase in sTfR level in the supplemented group and to a significant decrease in this parameter in the controls. Noticeably, athletes from the supplemented group presented with significantly higher pre-exercise levels of sTfR than the controls during the post-intervention ergometric test (Fig. 3d). Neither supplementation with POM nor physical exercise had a significant effect on serum ferritin levels in our study subjects (Fig. 3c), which is consistent with the results of other studies [40, 41].

## Conclusions

This study showed that the administration of pomegranate fruit juice, a dietary supplement with established high antioxidant potential, boosted the TAC of the study athletes, but had no significant effect on inflammatory markers or other parameters analysed. In the case of well-trained athletes, the training phase and adaptation to exercise loads also seem to be important determinants, but this hypothesis needs to be verified by further comprehensive studies.

## Abbreviations

ANOVA: Analysis of variance
Fe: Serum iron
IL - 1β: Interleukin 1 beta
IL-6: Interleukin
LA: Lactic acid
LIP: Labile iron pool
POM: Pomegranate juice
SD: Standard deviations
sTfR: Soluble transferrin receptor
TAC: Total antioxidant capacity
TIBC: Total iron binding capacity
TNF-α: Tumour necrosis factor alpha
UIBC: Unsaturated iron-binding capacity
